# Mitochondria: one of the vital hubs for molecular hydrogen’s biological functions

**DOI:** 10.3389/fcell.2023.1283820

**Published:** 2023-11-07

**Authors:** Xiaoyue Zhang, Fei Xie, Shiwen Ma, Chen Ma, Xue Jiang, Yang Yi, Yifei Song, Mengyu Liu, Pengxiang Zhao, Xuemei Ma

**Affiliations:** ^1^ Faculty of Environment and Life, Beijing University of Technology, Beijing, China; ^2^ Beijing Molecular Hydrogen Research Center, Beijing, China

**Keywords:** molecular hydrogen (H_2_), mitochondria, complex I, hydrogenase, mitochondrial quality control

## Abstract

As a novel antioxidant, a growing body of studies has documented the diverse biological effects of molecular hydrogen (H_2_) in a wide range of organisms, spanning animals, plants, and microorganisms. Although several possible mechanisms have been proposed, they cannot fully explain the extensive biological effects of H_2_. Mitochondria, known for ATP production, also play crucial roles in diverse cellular functions, including Ca^2+^ signaling, regulation of reactive oxygen species (ROS) generation, apoptosis, proliferation, and lipid transport, while their dysfunction is implicated in a broad spectrum of diseases, including cardiovascular disorders, neurodegenerative conditions, metabolic disorders, and cancer. This review aims to 1) summarize the experimental evidence on the impact of H_2_ on mitochondrial function; 2) provide an overview of the mitochondrial pathways underlying the biological effects of H_2_, and 3) discuss H_2_ metabolism in eukaryotic organisms and its relationship with mitochondria. Moreover, based on previous findings, this review proposes that H_2_ may regulate mitochondrial quality control through diverse pathways in response to varying degrees of mitochondrial damage. By combining the existing research evidence with an evolutionary perspective, this review emphasizes the potential hydrogenase activity in mitochondria of higher plants and animals. Finally, this review also addresses potential issues in the current mechanistic study and offers insights into future research directions, aiming to provide a reference for future studies on the mechanisms underlying the action of H_2_.

## 1 Introduction

Molecular hydrogen (H_2_) has long been considered as a physiologically inert gas until the 2007 discovery that H_2_ can protect the brain against ischemia-reperfusion (I/R) injury by selectively scavenging hydroxyl radicals and peroxynitrite (Ohsawa et al., 2007). This report unveiled the prelude of the research on H_2_ therapy, which has now become a burgeoning field of research in health and medical sciences. More than 2000 studies on the biological effects of H_2_ including over 100 clinical trials have been published over the last 15 years (LeBaron et al., 2023). The most commonly used hydrogen intervention methods in clinical trials are as follows: 1) inhaling hydrogen gas (2%–4%) or a hydrogen-oxygen mixture (66% H_2_/33% O_2_) twice a day, for 1–2 h each time. 2) drinking hydrogen-rich water (HRW) daily, ranging from 320 mL to 2 L 3) intravenous injection of hydrogen-rich saline (HRS), typically 500 mL per day. The duration of hydrogen intervention varies based on the specific disease type and the observation period intended by the researchers, spanning from 3 days to 2 years. These studies demonstrated the potential therapeutic effects in over 170 different human and animal disease models, including neurodegenerative diseases, metabolic diseases, inflammatory diseases, mitochondrial diseases, cancer, etc. (Ichihara et al., 2015). However, so far, the primary targets and the mechanism underlying the action of H_2_ remain largely unknown. Although the antioxidant hypothesis of scavenging hydroxyl radicals can help explain some biological effects of H_2_, due to the considerably lower reaction rate of H_2_ with hydroxyl radicals than other cellular antioxidants, this hypothesis has been debated for a long time. In addition to its antioxidant role, H_2_ has also been reported to exert multiple effects, such as anti-inflammation, anti-apoptosis, anti-shock, anti-dysmetabolism, activation of autophagy, preservation of mitochondrial function, etc. (Ichihara et al., 2015; Chen et al., 2021b; Slezak et al., 2021). Obviously, the extensive biological effects of H_2_ cannot be fully explained by its radical scavenging properties. There is thus a long way to go to thoroughly elucidate the mechanisms of action of H_2_.

In the process of exploring the potential mechanisms of H_2_ action, mitochondria have attracted mounting attention. As the “powerhouses of cell”, mitochondria are not only a source of ATP production, but are also involved in other cellular essential functions, including Ca^2+^ signaling, generation and modulation of reactive oxygen species (ROS), apoptosis, proliferation, lipid trafficking, etc. (Silwal et al., 2020; Brillo et al., 2021). Mitochondrial dysfunction and oxidative stress are implicated in numerous diseases, including heart disease, neurodegenerative disorders, metabolic diseases, and cancer (Bhatti et al., 2017; Peoples et al., 2019; Luo et al., 2020; Picca et al., 2020). The first proposed and widely accepted mechanism of H_2_ action is through selectively scavenging free radicals, which are mainly generated from the electron transport chain by electron escape during adenosine triphosphate (ATP) production in mitochondria. Due to its special properties, such as small size, low mass, neutral charge, nonpolarity, and high rate of diffusion, H_2_ has been suggested to rapidly penetrate cellular biomembranes and reach subcellular compartments (within 1 min) (Ohta, 2015; Gong et al., 2022). Although there is currently no direct evidence of uptake of H_2_ by mitochondria, growing evidence indicates that H_2_ could affect mitochondrial function and exert biological effects through the mitochondrial pathways. In addition, hydrogenosomes, the organelles found in wide-ranging anaerobic eukaryotes that generate ATP via hydrogen-producing fermentations, have been considered as anaerobic forms of mitochondria (Muller et al., 2012). Mitochondrial complex I has been shown to be structurally related to [NiFe]-hydrogenase, a member of the hydrogenase family which can catalyze both the consumption and production of H_2_ (Marreiros et al., 2013). These evidences indicate the potential relationship between mitochondria and H_2_ metabolism. Furthermore, mitochondria also contain a significant amount of Fe-porphyrin-containing proteins such as cytochromes. There is existing experimental evidence suggesting that Fe-porphyrin may serve as a direct target of H_2_’s action (Jin et al., 2023). In addition to mitochondria, effects of H_2_ on other cellular organelles, such as the endoplasmic reticulum (ER), have also been reported. Many experimental findings suggest that H_2_ has a significant inhibitory effect on ER stress (Song et al., 2015; Wu et al., 2015; Gao et al., 2017). Considering the potential H_2_ targets within mitochondria, such as free radicals and Fe-porphyrin, as well as the fact that mitochondria are evolutionarily related to hydrogenosomes, and a significant amount of literature on the effects of H_2_ on mitochondria has been reported, the present review mainly focuses on the effects of H_2_ on mitochondrial function and the mitochondrial pathways underlying the biological effects of H_2_, and also provide insights into the relationship between mitochondria and H_2_ metabolism.

## 2 Effects of H_2_ on mitochondria under pathological and normal conditions

Mitochondrial dysfunction is known to be closely associated with a wide range of human pathological and physiological conditions, such as cardiovascular diseases, cancer, metabolic diseases, neurodegenerative diseases, cell senescence and aging (Johnson et al., 2021; Zhunina et al., 2021; Miwa et al., 2022). Numerous studies have provided evidence indicating that H_2_ has the ability to alleviate mitochondrial dysfunction induced by disease or external stress (Table 1).

**TABLE 1:** Effects of H_2_ on mitochondria under normal and pathological conditions.

Indexes	H_2_ + model group vs. model group	H_2_ + sham group vs. sham group
ATP production	↑ Liu et al. (2016), Li et al. (2017), Chen et al. (2018), Dong et al. (2018), Chen et al. (2019), Niu et al. (2020), Xie et al. (2021), Dumbuya et al. (2022), Lian et al. (2022), ↓ Fan et al. (2022), NSC Luo et al. (2022)	NSC Li et al. (2017), Dong et al. (2018), Niu et al. (2020), Xie et al. (2021), Dumbuya et al. (2022), Lian et al. (2022)
Mitochondrial respiratory function	↑ Liu et al. (2016), Shimada et al. (2016), Dong et al. (2018), Chen et al. (2019), Yan et al. (2019), Lian et al. (2022), Zhao et al. (2023)	NSC Dong et al. (2018), Lian et al. (2022)
mPTP opening	↓ Cui et al. (2014), Liu et al. (2016), Li et al. (2017), Chen et al. (2018)	NSC Li et al. (2017)
MMP	↑ Yu et al. (2011), Cui et al. (2014), Li et al. (2017), Wu et al. (2018b), Dong et al. (2018), Chen et al. (2019), Mo et al. (2019), Chen et al. (2021a), Xie et al. (2021), Dumbuya et al. (2022), Lian et al. (2022), Zhang et al. (2023), Zhao et al. (2023), ↓ Ishihara et al. (2020)	NSC Li et al. (2017), Dong et al. (2018), Xie et al. (2021), Dumbuya et al. (2022), Lian et al. (2022)
mtROS production	↓ Ren et al. (2016), Mo et al. (2019), Chen et al. (2021a), Dumbuya et al. (2022)	NSC Dumbuya et al. (2022)
Complex I activity	↑ Dong et al. (2018), Niu et al. (2020), Xie et al. (2021), Fan et al. (2022), Zhao et al. (2023)	NSC Dong et al. (2018), Niu et al. (2020), Xie et al. (2021)
Complex I protein expression	↓ Fan et al. (2022)	NA
Superoxide generation in complex I	↓ Ishihara et al. (2020)	NA
Complex II activity	NSC Dong et al. (2018), Xie et al. (2021), Fan et al. (2022), Zhao et al. (2023)	NSC Dong et al. (2018), Xie et al. (2021)
Complex II protein expression	NSC Fan et al. (2022)	NA
Complex III activity	↑ Niu et al. (2020), NSC Fan et al. (2022)	NSC Niu et al. (2020)
Complex III protein expression	NSC Fan et al. (2022)	NA
Superoxide generation in complex III	↓ Ishihara et al. (2020)	NA
Complex IV activity	↑ Fan et al. (2022)	NA
Complex IV protein expression	↑ Chaoqun et al. (2021), NSC Fan et al. (2022)	NA
Complex V activity	↑ Fan et al. (2022)	NA
Complex V protein expression	NSC Fan et al. (2022)	NA
mtDNA copy number	↑ Chen et al. (2019)	NA
mMDA, mGSSG levels	↓ Liu et al. (2016)	NA
mGSH levels	↑ Liu et al. (2016)	NA
mSOD, mCAT, mGpx activities	↑ Liu et al. (2016), Niu et al. (2020)	NA
MFN1 expression	↓ Fan et al. (2022)	NA
MFN2 expression	↑ Dong et al. (2018), Zhang et al. (2023), Zhao et al. (2023), NSC Fan et al. (2022)	NSC Dong et al. (2018)
OPA1 expression	↑ Zhang et al. (2023)	NA
Drp1 expression	↓ Dong et al. (2018), Lian et al. (2022), Zhang et al. (2023), Zhao et al. (2023), NSC Fan et al. (2022)	NSC Dong et al. (2018), Lian et al. (2022)
VDAC1 expression	↓ Mo et al. (2019)	NA
Citrate synthase activity	↑ Niu et al. (2020)	NSC Niu et al. (2020)
Na+-K+-ATPase activity	↑ Mo et al. (2019)	NA
Ca2+-Mg2+-ATPase activity	↑ Mo et al. (2019)	NA
Mitophagy	↑ Wu et al. (2018b), Chen et al. (2019), Yan et al. (2019), Chen et al. (2021a)	NA
Mitochondrial biogenesis	↑ Xie et al. (2021), Luo et al. (2022), Zhang et al. (2023)	NSC Xie et al. (2021)
mtUPR	⇑ Hasegawa et al. (2022)	↑ Sobue et al. (2017)
mitoKATPs	↑ Yoshida et al. (2012), Jiao et al. (2019), Zhang et al. (2021)	NA

Abbreviations: NSC, no significant change; mPTP, mitochondrial permeability transition pore; MMP, mitochondrial membrane potential; mtROS, mitochondrial reactive oxygen species; NA, not available; mtDNA, mitochondrial deoxyribonucleic acid; mMDA, mitochondrial malondialdehyde; mGSSG, mitochondrial glutathione disulfide; mGSH, mitochondrial glutathione; mSOD, mitochondrial superoxide dismutase; mCAT, mitochondrial catalase; mGpx, mitochondrial glutathione peroxidase; MFN1/2, mitofusin 1/2; OPA1, optic atrophy 1; Drp1, dynamin-related protein 1; VDAC1, voltage dependent anion channel 1; mtUPR, mitochondrial unfolded protein response; ⇑, not all types of cancer cell lines exhibited enhanced mtUPR after H2 intervention; mitoKATPs, mitochondrial ATP-sensitive potassium channel.

In most cases, compared to the disease or stress-induced model group, H_2_ treatment exhibited positive effects, including increased mitochondrial ATP production, enhanced mitochondrial respiration, inhibition of mPTP opening, improved MMP, reduced mtROS production, elevated complex I activity, activation of mitophagy, mitochondrial biogenesis, and mitoKATPs, *etc*. Although H_2_ has also been reported to enhance the activity of mitochondrial complex I, III, IV, and V in ovalbumin (OVA) sensitization and challenge-induced allergic airway inflammation mice (Niu et al., 2020) or lipopolysaccharide (LPS)-induced acute lung injury (ALI) mice (Fan et al., 2022), the current evidence is still relatively limited, highlighting the need for further research in this area. In a few cases, however, the impact of H_2_ on mitochondrial indicators seems to exhibit inconsistency among studies. Although H_2_ increased ATP production in most cases, Fan *et al.* reported a significant decrease in ATP production in lung tissue of mice with ALI following H_2_ treatment (Fan et al., 2022). This may be caused by the LPS-induced increase in neutrophils observed in ALI mice while H_2_ reversed the effect of LPS on ATP production. There are some inconsistencies between this study and other reports, such as a decrease in the expression of MFN1 induced by H_2_, while no significant effect is observed on the expression of MFN2 and Drp1. These inconsistencies warrant further investigation. Luo *et al.* examined the impact of H_2_ on exercise endurance and found that intake of HRW did not result in an increase in ATP production in skeletal muscle following high-intensity endurance exercise. One plausible explanation is that the skeletal muscle tissue samples were obtained immediately after exhaustive exercise, leading to depletion of ATP stores (Luo et al., 2022). Hasegawa et al. found that H_2_ has the potential to promote the proliferation of specific tumor cell types. Notably, in such tumor cells, H_2_ demonstrates a significant ability to activate the mtUPR response, likely attributed to the high basal and spare mitochondrial electron transport chain (ETC) activities of these types of tumor cells (Hasegawa et al., 2022). In addition, in terms of certain mitochondrial indicators, such as complex II activity and the expression of complex II, III and V, remain largely unchanged after hydrogen treatment in both the LPS-induced ALI (Fan et al., 2022) and septic mouse models (Dong et al., 2018; Xie et al., 2021; Zhao et al., 2023), which may be attributed to the fact that external stress did not significantly affect these indicators in the model group. In conclusion, H_2_ has a significant mitigating effect on altered mitochondrial function caused by external stress.

Moreover, there is initial clinical evidence suggesting a significant impact of H_2_ on mitochondria. Ito et al. performed an open-label and randomized double-blind placebo-controlled trial on the consumption of HRW in patients with mitochondrial myopathy (Ito et al., 2011). The results demonstrated a marked reduction in the serum lactate/pyruvate ratio after HRW intake, suggesting that H_2_ may alleviate mitochondrial ETC dysfunction through its antioxidative effects or other unknown signaling pathways. Additionally, Akagi et al. found that H_2_ inhalation can restore exhausted CD8^+^ T cells in lung cancer patients (Akagi and Baba, 2020), a phenomenon also observed in colorectal cancer (Akagi and Baba, 2019). They speculated that this may be attributed to H2 activating mitochondria, as the levels of CoQ10, a mitochondrial function marker, significantly increased in the serum and were significantly correlated with prognosis. Currently, clinical evidence regarding the impact of H_2_ on mitochondria remains relatively limited, and future clinical investigations can further examine the role of H_2_ from a mitochondrial perspective.

Despite extensive documentation of the impact of H_2_ on mitochondria under disease or external stress conditions, there is relatively limited research on the effects of H_2_ on mitochondria in normal physiological conditions. Based on the currently available evidence, it seems that H_2_ does not have a significant impact on the majority of mitochondrial indicators, with the exception of mtUPR activation (Sobue et al., 2017). Sobue *et al.* found that the expression of mtUPR-related genes was significantly increased in H_2_-treated mice liver in non-stressed normal conditions, indicating that H_2_ can activate the mtUPR response under normal physiological conditions (Sobue et al., 2017). However, the evidence for the activating effect of H_2_ on mtUPR response is still very limited, further investigations should provide more evidence on the effects of H_2_ on mtUPR response and other mitochondrial indicators in the non-stressed normal conditions.

## 3 Mitochondrial pathways underlying the biological effects of H_2_

**FIGURE 1:**
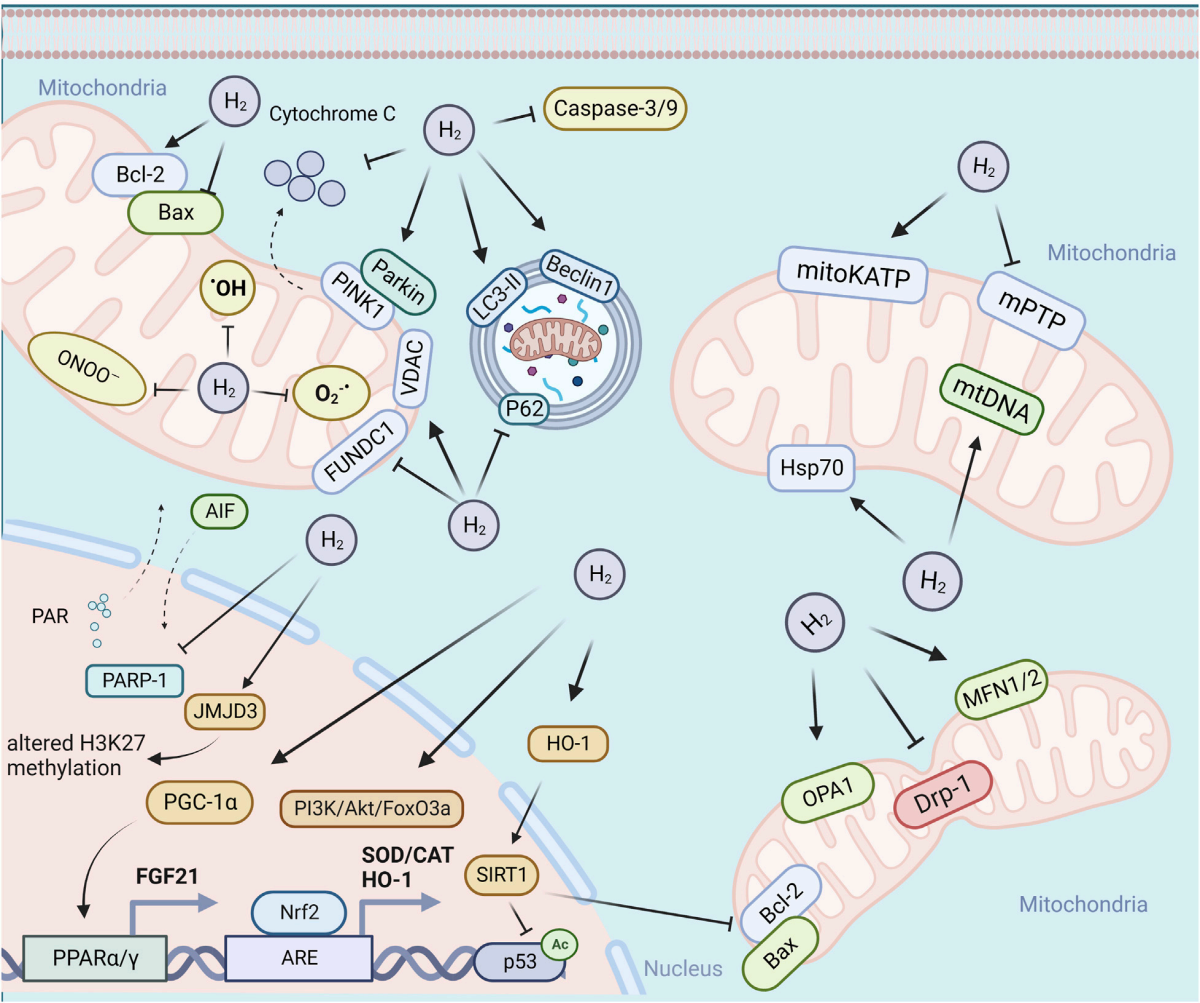
H_2_ attenuate mitochondrial damage by modulating mitochondrial quality control through diverse pathways. Abbreviations: PARP-1, poly (ADP-ribose) polymerase-1; PINK1, PTEN induced putative kinase 1; LC3-II, microtubule-associated protein light chain 3-II; PGC-1, peroxisome proliferator-activated receptor-gamma (PPARγ) co-activator-1alpha; TFAM, mitochondrial transcription factor A; NRF1/2, erythroid 2-related factor 1/2; PPARγ, peroxisome proliferator-activated receptor-gamma.

Mitochondria, being highly vulnerable to injury or damage, primarily rely on two types of quality control mechanisms to maintain their homeostasis, including molecular quality control mechanisms (such as antioxidant defense, protein quality control, and mtDNA repair), and organelle quality control mechanisms (such as mitochondrial dynamics, mitophagy, and mitochondrial biogenesis) (Tang et al., 2020). The loss of mitochondrial quality control can trigger mitochondrial damage and dysfunction, ultimately leading to cell death, tissue injury, and the potential onset of organ failure. As shown in Figure 1, H_2_ has been demonstrated to effectively attenuate mitochondrial damage by modulating mitochondrial quality control through diverse pathways in response to varying degrees of mitochondrial damage. The following section will address the various pathways implicated in the mitigation of mitochondrial damage by H_2_.

### 3.1 Reducing mtROS

The antioxidant effect of H_2_ is the earliest proposed hypothesis of its mechanism of action. As the main source of cellular ROS, mitochondria have been considered as a key battlefront in combating the free radicals by H_2_. The present experimental evidence indicates that H_2_ may exert antioxidant effects in at least three ways: 1) direct scavenging of free radicals. Ohta’s group provided the first evidence that H_2_ may play an antioxidant role by selectively scavenging hydroxyl radicals and peroxynitrite (Ohsawa et al., 2007), which has been challenged due to the lower reaction constant between hydroxyl radicals and H_2_ than those of other radical scavengers, low H_2_ dosage and short dwell time in cell. 2) inhibition of mtROS generation. The inhibitory effect of H_2_ on mtROS production has been previously reported (Chen et al., 2019; Chen et al., 2021a; Dumbuya et al., 2022), however, these studies measured mtROS production by using edichloro-dihydro-fuorescein diacetate (DCFH-DA), which cannot cross the inner mitochondrial membrane (Hasegawa et al., 2022), thus it cannot truly reflect changes in ROS levels within mitochondria. According to the theory of “frustrated Lewis pair” (FLP), Ishibashi proposed that H_2_ might be activated in the Q-chamber of mitochondria, providing electrons and protons to the ubiquinone (UQ) species, preventing the electron leakage from the ETC and ultimately inhibiting ROS production (Ishibashi, 2019). Ishihara et al. provided evidence that H_2_ mainly suppress superoxide generation in complex I, which may be caused by H_2_ induced reduction in MMP (Ishihara et al., 2020). Some other studies also provided evidence of the inhibition of mitochondrial superoxide production by H_2_, however, these studies may have limitations with respect to the specificity of mitochondrial superoxide detection. For example, one study used MitoSOX as a fluorescent mitochondrial superoxide indicator with high concentrations (5 mM) (Mo et al., 2019). For one hand, MitoSOX can form two products, namely ethidium (E^+^), a non-specific oxidation product, and the superoxide-specific adduct 2-hydroxyethidium (2-OH-E^+^). The two products have overlapping fluorescence spectra, it is therefore difficult to accurately measure only 2-OH-E^+^ by using simple fluorescence-based microscopic assays (Murphy et al., 2022). On the other hand, high concentrations of MitoSOX (exceeding 2 µM) can result in substantial accumulation of mitoSOX in cytoplasm and thus can reduce the specificity of mitochondrial superoxide detection (Dikalov and Harrison, 2014). Thus, more experimental evidence is required to support the hypothesis of the inhibition of superoxide production by H_2_. 3) enhancement of the cellular antioxidative capability. It has been reported that H_2_ could increase antioxidant enzymes, including SOD, CAT, Gpx, heme oxygenase-1 (HO-1), and NAD(P)H quinone dehydrogenase 1 (NQO1) (Liu et al., 2016; Li et al., 2019). The activation of Nrf2-mediated antioxidant signaling pathway by H_2_ may be responsible for the increased expression of the antioxidant enzymes (Li et al., 2019; Zheng et al., 2021; Han et al., 2023). In conclusion, available evidence indicates the inhibitory effect of H_2_ on mtROS, which may be mediated by directly scavenging free radicals, inhibiting free radical production or enhancing cellular antioxidant capacity, although the detailed mechanism needs to be further investigated.

### 3.2 Regulation of apoptosis

Mitochondria play a central role in the induction of apoptosis, a form of programmed cell death (PCD), which is essential for maintaining normal cellular homeostasis and is governed by levels of anti-apoptotic and pro-apoptotic proteins (Dadsena et al., 2021). It has been extensively reported that H_2_ can inhibit apoptosis, as demonstrated by the upregulation of the anti-apoptotic factor B-cell lymphoma 2 (Bcl-2), downregulation of the pro-apoptotic factor Bcl-2-associated X protein (Bax), inhibition of cytochrome c release and the translocation of Bax to mitochondria, suppression of the expression or activity of caspases, as well as decreased TUNEL (terminal deoxynucleotidyl transferase dUTP nick end labeling) staining positive cells or Annexin V-positive/propidium iodide (PI)-negative staining cells (Cui et al., 2014; Liu et al., 2016; Li et al., 2017; Jiao et al., 2019; Mo et al., 2019; Adzavon et al., 2022; Dumbuya et al., 2022). Oxidative stress has been implicated to cause apoptosis via both the mitochondria-dependent and mitochondria-independent pathways (Sinha et al., 2013). There are reportedly at least three oxidative stress mediated mitochondria-dependent pathways involved in the inhibition of apoptosis by H_2_. First, H_2_ has been demonstrated to inhibit high glucose-mediated oxidative stress induced apoptosis of Schwann cells via suppression of the activation of the DNA nick sensor enzyme PARP-1, which can be activated by oxidative stress-induced DNA breakage and induce either caspase-independent apoptosis via translocation of apoptosis-inducing factor (AIF) from mitochondria to the nucleus or caspase-dependent apoptosis by activation of caspase-3 (Yu et al., 2015). Second, H_2_ could suppress apoptosis through Nrf2/antioxidant responsive element (ARE) pathway (Li et al., 2018; Li et al., 2019). H_2_-induced Nrf2 activation can further stimulate the expression of ARE-responsive genes, including HO-1, followed by upregulation of NAD-dependent protein acetylase Sirtuin1 (SIRT1), which in turn can suppress Bax expression while reducing p53 acetylation levels, decreasing caspase-3 cleavage, and ultimately inhibiting apoptosis. In addition, Mo et al. (2019) proposed that H_2_-induced upregulation of Bcl-2, as another downstream target of Nrf2/ARE pathway, was responsible for the inhibition of apoptosis. In the presence of apoptotic signals, the tightly binding of anti-apoptotic Bcl-2 could close VDAC1, a vital component of mPTP, to prevent cytochrome c release, and untimately inhibit apoptosis (Tsujimoto and Shimizu, 2000). Third, the phosphatidylinositol 3′-kinase (PI3K)/Akt/forkhead box O3 (Foxo3a) signaling pathway may also be involved in the inhibitory effect of H_2_ on apoptosis. Wu et al. provided evidence that H_2_ could protect type II alveolar epithelial cells from hyperoxia-induced apoptosis via activation of PI3K/Akt/Foxo3a pathway (Wu et al., 2018a).

In most cases, H_2_ exhibits an inhibitory effect on apoptosis, however, H_2_ has also been found to promote apoptosis in some types of cancers, including colon cancer and lung cancer (Runtuwene et al., 2015; Wang et al., 2018; Liu et al., 2020; Zan et al., 2022), but not in all cancers, such as liver cancer (Runtuwene et al., 2015). In addition to cancer cells, the promoting effect on apoptosis has also been observed in vascular smooth muscle cells (VSMCs) (Chen et al., 2013). These studies proposed the potential mechanisms underlying the pro-apoptotic effect of H_2_, such as activating AMP-activated protein kinase (AMPK) pathway (Runtuwene et al., 2015), down-regulating structural maintenance of chromosomes 3 (SMC3) (Wang et al., 2018), inhibiting the activation of the signal transducer and activator of transcription 3 (STAT3)/Bcl2 signaling pathway (Liu et al., 2020), up-regulating the tumor suppressor protein P53 (Zan et al., 2022), and inactivating the Ras-extracellular-regulated protein kinases 1/2 (ERK1/2)-mitogen-activated or extracellular signal-regulated protein kinase kinases 1 and 2 (MEK1/2) and Akt pathways (Chen et al., 2013). In conclusion, H_2_ exhibits either anti-apoptotic or pro-apoptotic effect, which may be at least determined by the cell type and cellular environment.

### 3.3 Regulation of mitochondrial dynamics

As highly plastic and interconnected organelles, mitochondria dynamically balance between fusion and fission, and degradation of damaged mitochondria by mitophagy, which together maintain mitochondrial homeostasis and contribute to key cellular pathways (Ma et al., 2020). The term mitochondrial dynamics involves continuous fission and fusion, selective degradation, and transport (Chan, 2020). Disturbed mitochondrial dynamics leads to decreased ATP production and mitochondrial DNA mutation, which ultimately leads to cell death (Liu et al., 2022). Mitochondrial fission is essential for segregation of mitochondria into daughter cells during mitosis and removal of damaged or dysfunctional components of mitochondria via mitophagy, and is mediated by recruitment of cytosolic Drp1 and its receptors on the outer mitochondrial membrane (OMM) (Tang et al., 2020). Mitochondrial fusion, mediated by MFN1/2 and OPA1, enables the exchange of gene products and metabolites between damaged and healthy mitochondria to alleviate organelle stress and prevent mitochondrial elimination via mitophagy (Tang et al., 2020). The net balance between fission and fusion dynamically regulates the number and morphology of mitochondria (Chan, 2020). Previous studies have provided both *in vivo* and *in vitro* evidence that H_2_ could alleviate lipopolysaccharide (LPS) or cecal ligation and puncture (CLP)-induced mitochondrial fission and promote mitochondrial fusion, evidenced by downregulation of Drp1 expression and upregulation of MFN1/2 or OPA1 expression (Dong et al., 2018; Lian et al., 2022; Zhang et al., 2023; Zhao et al., 2023). Lian et al. showed that H_2_ had a similar inhibitory effect to Mdivi-1, a mitochondrial fission blocker, on LPS-induced upregulation of Drp1 and its recruitment to mitochondria, which further confirmed the suppressing role of H_2_ on LPS-induced mitochondrial fission (Lian et al., 2022). Mitochondrial fragmentation are often associated with mitochondrial dysfunction, and elongated mitochondria are thought to be more bioenergetically efficient (Chan, 2020). The regulation of H_2_ on mitochondrial fission and fusion is consistent with the improvement of mitochondrial function. Although it has been reported that the H_2_-induced upregulation of HO-1 expression may be responsible for the inhibition of stress-induced mitochondrial fission, the detailed molecular mechanisms underlying the regulation of mitochondrial fission/fusion by H_2_ remains unclear. Mitochondrial fission and fusion proteins can be regulated by various post-translational modifications (PTMs), including phosphorylation, O-GlcNAcylation, acetylation, ubiquitination, SUMOylation, etc., which facilitate rapid responses to stress conditions (Sabouny and Shutt, 2020). Further research on the potential PTMs of mitochondrial fission/fusion proteins would aid in elucidating the mechanism underlying the effects of H_2_ on mitochondrial dynamics. In addition, most of the current studies only checked the expression pattern of mitochondrial fission/fusion proteins in septic models, further study should be performed to quantify the extent of mitochondrial fission and fusion and identify the type of fission (midzone or peripheral fission) and fusion (transient or complete fusion) under other stressful conditions (Qin and Xi, 2022).

Mitochondrial autophagy, also known as mitophagy, is a cellular process that selectively identify and degrade the damaged or dysfunctional mitochondria via the specific sequestration and engulfment by autophagosomes for subsequent lysosomal degradation (Ma et al., 2020). In general, mitophagy can be divided into ubiquitin (Ub)-dependent mitophagy and Ub-independent or receptor based mitophagy, although other forms of mitophagy also exist, such as lipid based mitophagy and micromitophagy (Choubey et al., 2022; Lu et al., 2023). Ub-dependent mitophagy relies on extensive ubiquitination of mitochondrial surface proteins to initiate mitophagy. Among the Ub-dependent pathways, the PINK1/Parkin pathway is currently the best-studied (Lu et al., 2023). Previous studies provided evidence that H_2_ could further enhance stress-induced mitophagy, as demonstrated by upregulation of Beclin1, PINK1, Parkin, and VDAC, enhancing LC3-I conversion to LC3-II, and downregulation of P62 (Wu et al., 2018b; Chen et al., 2019; Yan et al., 2019; Chen et al., 2021a; Zhang et al., 2023). The PINK1/Parkin pathway may be involved in the promotion of mitophagy by H_2_ (Wu et al., 2018b; Chen et al., 2021a). In addition, as one of the most prominent mitophagy receptors, FUN14 domain containing 1 (FUNDC1) regulates receptor-mediated mitophagy independently of the PINK1/Parkin-dependent pathway through PTMs, including ubiquitination and phosphorylation (Choubey et al., 2022). It has been shown that H_2_ could further enhance sepsis-induced elevation of FUNDC1 expression, and the FUNDC1 inhibitor peptide P could effectively reverse the protective effect of H_2_, indicating the involvement of FUNDC1 in the stimulation of mitophagy by H_2_ (Yan et al., 2019). Future research should further explore the potential role of H_2_ on PTMs of FUNDC1.

The subcellular localization of mitochondria, as a result of a combination of transport along microtubule tracks and anchoring on actin filaments, is essential for maintaining cell polarity, morphology, and cellular homeostasis (Furnish and Caino, 2020). There is growing evidence linking abnormal mitochondrial transport to mitochondrial dysfunction and metabolic alterations in a variety of diseases, including neurological diseases, diabetes, and cancer (Furnish and Caino, 2020). Previous studies have demonstrated that the calcium-induced conformational changes and PTMs mediated by kinases, GTPases, and E3 ubiquitin ligases, and some signaling pathways, such as PINK1/Parkin and MAPK/p38 pathway, are involved in the regulation of mitochondrial transport (Furnish and Caino, 2020). Considering the regulation of mitochondrial function and the PINK1/Parkin and MAPK/p38 pathway by H_2_, it is likely that H_2_ might exert regulatory effect on mitochondrial transport, although this needs further investigation.

In conclusion, the current studies demonstrated the regulatory role of H_2_ on mitochondrial dynamics, including inhibition of mitochondrial fission, promotion of mitochondrial fusion, and further enhancement of stress-induced mitophagy.

### 3.4 Promotion of mitochondrial biogenesis

The process of mitochondrial biogenesis involves the generation of new mitochondria from existing ones, and it can be translationally regulated by PGC-1α. Dysregulated mitochondrial biogenesis has been implicated in senescence and ageing, as well as the initiation and progression of metabolic diseases, neurodegeneration and cancer (Popov, 2020). The activation of PGC-1α can occur through phosphorylation by AMPK or de-acetylation by SIRT1, which further triggers a cascade of events that ultimately induces mitochondria biogenesis. This process involves the activation of NRF-1 and NRF-2, which in turn activate TFAM. The activation of this PGC-1α/NRF-1/2/TFAM pathway leads to the synthesis of mitochondrial DNA and proteins and generation of new mitochondria (Popov, 2020). The promotion of mitochondrial biogenesis by H_2_ has been previously reported, as demonstrated by increased expression of mtDNA (mtCoxII, mtCoxIV, and mtNd1) and mitochondrial biogenesis-related genes (Pgc-1α, Tfam, Nrf-1/2, and Pparγ) (Chaoqun et al., 2021; Xie et al., 2021; Luo et al., 2022; Zhang et al., 2023). The H_2_-induced mitochondrial biogenesis may be mediated by PPARγ/AMPK/SIRT1-PGC-1α-NRF1/2-TFAM pathway (Chaoqun et al., 2021; Xie et al., 2021; Luo et al., 2022). In addition to the regulation at transcriptional level, mitochondrial biogenesis can also be governed by post-translational processes, such as mitochondrial protein import regulation (Devaux et al., 2010). The effect of H_2_ on the post-translational regulation of mitochondrial biogenesis needs to be further investigated.

### 3.5 Activation of the mitochondrial unfolded protein response

The mtUPR is an evolutionarily conserved defense mechanism that maintains mitochondrial proteostasis by inducing the expression of mitochondrial chaperones and proteases in response to diverse forms of mitochondrial stress from impaired protein translation to mtDNA defects (Naresh and Haynes, 2019). A growing body of evidence suggests that mtUPR plays an important role in a variety of human diseases, particularly aging-related neurodegeneration, as well as metabolic diseases, heart diseases and cancers (Zhu et al., 2021a; Zhu et al., 2021b; Wodrich et al., 2022). The canonical signaling pathway of the mammalian mtUPR is primarily mediated by phosphorylation of eukaryotic translation initiation factor 2 subunit 1 (eIF2α). Although eIF2α molecules are constitutively phosphorylated, excessive ROS will further increase the level of eIF2α phosphorylation by four stress-responsive kinases, leading to the reduction in global protein synthesis and selective translation of mRNAs with open reading frames in the 5′ untranslated region (5′UTR), including C/EBP homologous protein (CHOP), transcription factor 4 (ATF4) and ATF5 (Shpilka and Haynes, 2018). In addition to transcriptional regulation, epigenetic regulation by chromatin remodeling also plays an important role in mtUPR activation, and the histones (H3K9, H3K27) and demethylases (JMJD-3.1/JMJD3) are involved in this process (Wodrich et al., 2022). Previous studies have demonstrated that H_2_ could induce beneficial effects through activation of mtUPR. Sobue *et al.* provided evidence that H_2_ can counteract dietary stress probably by activation of mtUPR. This process may be mediated by altering H3K27 methylation status via inducing JMJD3 expression, which in turn upregulates the expression of mitochondrial chaperone mitochondrial heat shock protein 70 (mtHsp70) and other mtUPR-related genes and ultimately activates mtUPR (Sobue et al., 2017). Furthermore, Hasegawa *et al.* reported the proliferation-promoting effects of H_2_ on four out of seven human cancer cell lines. Their further study found that H_2_ can activate the mtUPR in these responders, which share the common feature of high basal and spare mitochondrial ETC activities (Hasegawa et al., 2022). In contrast to the decline in mtUPR in the development of neurodegenerative diseases, the activation of the mtUPR is present in most types of cancer, and its inhibition could alleviate cancer invasion (Cilleros-Holgado et al., 2023). Hasegawa et al.‘s research reminds us that extensive and scrutinized pre-clinical and clinical studies are needed to elucidate the beneficial and adverse effects of H_2_ on various types of cancer. Additionally, the involvement of cell non-autonomous induction of mtUPR has also been reported. In mammals, many mitochondrial stresses can be signaled to the distal tissues to induce mtUPR through the secretion of mitokines, such as fibroblast growth factor 21 (FGF21) (Wodrich et al., 2022). Kamimura *et al.* found that H_2_ could alleviate dietary stress and stimulate energy metabolism probably by upregulation of FGF21 expression, although the involvement of mtUPR was not analyzed (Kamimura et al., 2011). The possible cell non-autonomous mtUPR induced by H_2_ need to be further investigated. In conclusion, the current research provides experimental evidence demonstrating that H_2_ can activate the mtUPR in response to mitochondrial stress. However, additional studies are necessary to explore whether H_2_ elicits similar effects under various stress conditions and in diverse cell types. Furthermore, further investigation is required to uncover the molecular mechanisms responsible for the activation of mtUPR by H_2_.

### 3.6 Activation of the mitochondrial ATP-sensitive potassium channels

MitoKATPs, located in the IMM, is a form of potassium channel that is activated by both GTP and GDP, and inhibited by ADP and long-chain acyl-CoA esters (Pereira and Kowaltowski, 2021). It has been extensively reported that the pharmacological activation of mitoKATPs could promote the entry of potassium ions into the mitochondrial matrix and provide protection against I/R injury (Laskowski et al., 2019). Recent studies have shown that H_2_ is likely to exert protective effects against cardiac and neurological disorders, including myocardial infarction (Yoshida et al., 2012), diabetic peripheral neuropathy (Jiao et al., 2019), and subarachnoid hemorrhage (Zhang et al., 2021), via activating the mitoKATP pathway, evidenced by the abolishment of cardioprotective or neuroprotective effects of H_2_ when co-treatment with 5-hydroxydecanoate (5-HD, a selective mitoKATP blocker). Yoshida et al. (2012) proposed that H_2_-induced cardioprotection conferred by activating mitoKATP might be mediated by the inhibition of mPTP, since the infarct size-limiting effect of H_2_ was abrogated in the presence of atractyloside (Atr, a mPTP opener). The mPTP is non-specific, voltage- and Ca^2+^-dependent high-conductance channel located in the IMM and mediated the mitochondrial permeability transition (mPT), which allows for the transit of solutes with molecular masses up to 1,500 Da, including Ca^2+^, metabolic substrates and ATP, across the IMM (Wacquier et al., 2020). The I/R-induced mPTP opening not only causes mitochondria to produce high levels of cytotoxic ROS, but also consumes ATP to maintain Δψm, which further causes cellular damage (Zorov et al., 2009). The activation of mitoKATP has been reported to inhibit mPT via ROS activation of protein kinase C-ε (PKC-ε), since the mPT inhibition could be blocked by ROS scavenger (N-2-mercaptopropionylglycine, MPG) and PKC inhibitors (Costa et al., 2006). In addition to ROS, many regulatory factors are involved in the regulation of mPT, including Ca^2+^, proteins, lipids, etc. (Zorov et al., 2009), and further research is needed to investigate whether H_2_ can inhibit mPT through these regulatory factors. Additionally, the opening of mitoKATP by H_2_ can also activate the downstream ERK1/2 and MAPK/p38 signaling pathways, thus exerting an anti-pyroptosis effect (Zhang et al., 2021). Besides the activation of mitoKATP, whether H_2_ can also activate other mitochondrial K^+^ channels, and whether H_2_ affects the transport of other mitochondrial ions (e.g., Ca^2+^, Na^+^, etc.) needs further investigation.

### 3.7 Stimulation of mitochondrial bioenergetics

Under conditions of disease or stress, prolonged mitochondrial damage leads to impaired energy metabolism, resulting in diminished ATP production and elevated ROS burden. H_2_ has been demonstrated to stimulate mitochondrial bioenergetics under pathological or external stress, as evidenced by preserved MMP and increased ATP production (Table 1). Ostojic proposed four possible mechanisms for H_2_ to regulate mitochondrial bioenergetics through gene-expression alterations, including: 1) upregulation of growth hormone (GH) secretagogue receptor 1α (GHS-R1α) through the involvement of ghrelin, a growth hormone secretagogue; 2) activation of glucose transporter 1 (GLUT1) through a ghrelin-related pathway; 3) activation of GLUT4 through a pathway independent of ghrelin; and 4) enhanced expression of FGF21 through a pathway independent of ghrelin (Ostojic, 2017). Furthermore, H_2_ can stimulate mitochondrial biogenesis by enhancing the activity of enzymes involved in the mitochondrial tricarboxylic acid (TCA) cycle, mitochondrial respiratory chain and oxidative phosphorylation, including complex I, III, IV, V, as well as citrate synthase (Table 1). However, the exact mechanism by which H_2_ affects the activity of these mitochondrial enzymes, whether it is through direct or indirect action, remains uncertain.

**FIGURE 2:**
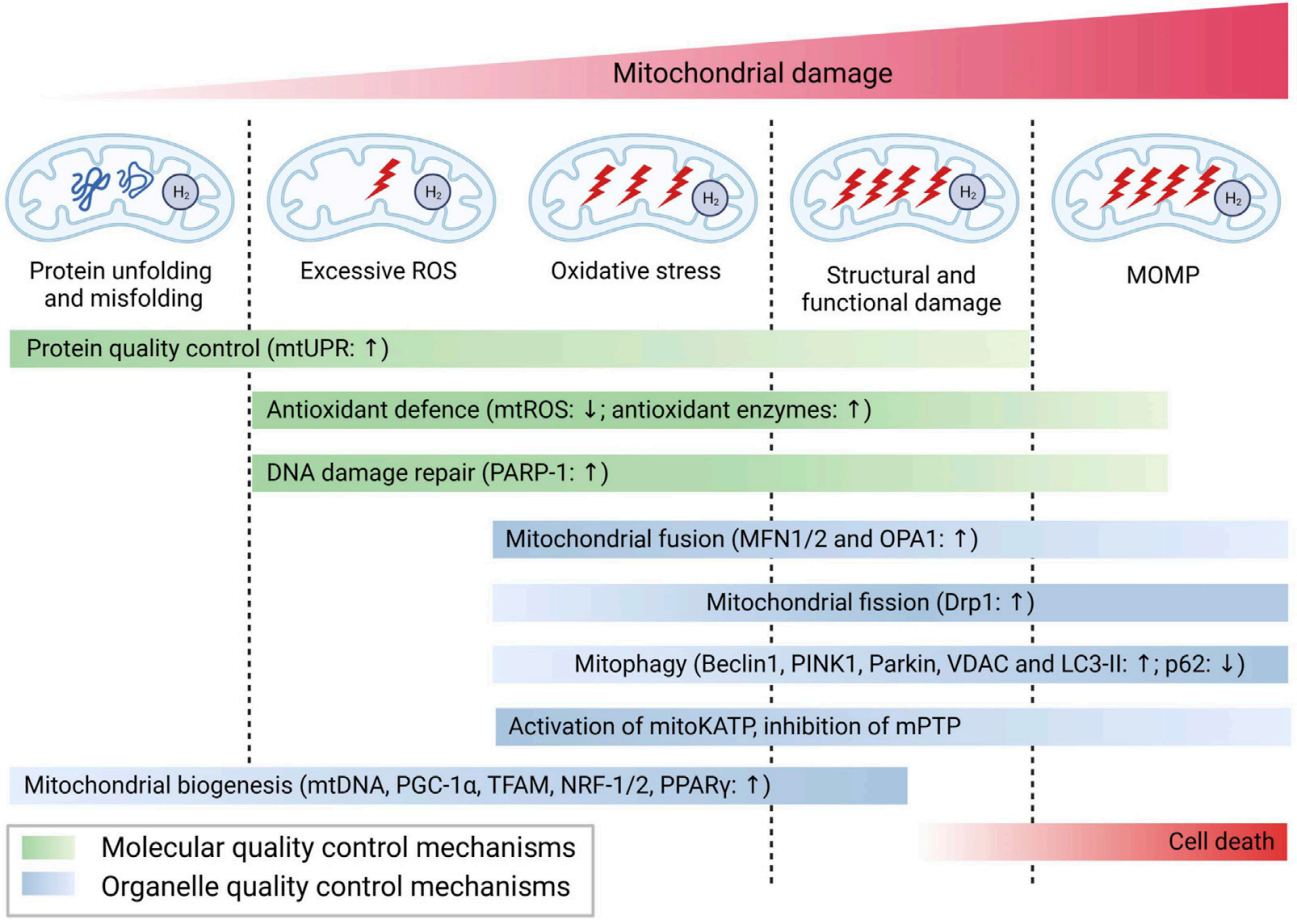
Possible mechanisms for the biological role of H_2_ via mitochondrial pathways.

In conclusion, in response to different degree of mitochondrial damage, H_2_ could alleviate mitochondrial dysfunction through diverse mechanisms of action. The specific molecular mechanisms are summarized in Figure 2.

## 4 H_2_ metabolism in eukaryotic organisms and its relationship with mitochondria

### 4.1 Mitochondria and related organelles possess the capacity to generate H_2_

Mitochondria are widely recognized for their crucial role in ATP production through aerobic respiration. In oxygen-limited environments, mitochondria undergo remarkable adaptations, characterized by reductive modifications in both structure and function. These adaptations involve a fusion of characteristics inherited from aerobic mitochondria with lineage-specific components and mechanisms, resulting in the emergence of a diverse range of organelles commonly referred to as mitochondrion-related organelles (MROs), which are found in a wide range of anaerobic/microaerophilic eukaryotes (Wani et al., 2021). As shown in Table 2, Muller et al. (2012) proposed a detailed classification of MROs, which consists of five distinct classes based on energy metabolism: aerobic mitochondria, anaerobic mitochondria, H_2_-producing mitochondria, hydrogenosomes, and mitosomes. Among these MROs, both H_2_-producing mitochondria and hydrogenosomes exhibit H_2_-producing activity. H_2_-producing mitochondria, a type of MROs found in organisms like Nyctotherus ovalis and Blastocystis sp., possess a membrane-associated ETC that utilizes protons as electron acceptors, generating ATP through substrate-level phosphorylation via anaerobic pyruvate metabolism and H_2_ production rather than chemiosmotic mechanisms (Roger et al., 2017). Hydrogenosomes, the H_2_-producing mitochondrial homologs found in some anaerobic microbial eukaryotes, function as energy-generating MROs through fermentative metabolism of pyruvate, while lacking typical mitochondrial features such as ETC and mitochondrial genome (Shiflett and Johnson, 2010). Similar to H_2_-producing mitochondria, hydrogenosomes produce ATP solely through substrate-level phosphorylation. Hydrogenases, as a group of versatile metalloenzymes, have the ability to catalyze the reversible conversion of H_2_ by both splitting and formation, and they can be classified into three types (FeFe, NiFe, and Fe-hydrogenase) based on the metal content in their active sites (Lubitz et al., 2014). It has been established that both H_2_-producing mitochondria and hydrogenosomes use FeFe-hydrogenase to catalyze the evolution of H_2_. Pyruvate: ferredoxin oxidoreductase (PFO), a prevalent enzyme found in anaerobic eukaryotic microbes, collaborates with FeFe-hydrogenase to preserve redox balance by transferring electrons from food oxidation through ferredoxin (Fd) to protons, resulting in the production of H_2_ as a byproduct (Gould et al., 2019). In addition to the MROs in anaerobic/microaerophilic eukaryotes, Ma’s group’s recent study presented compelling evidence supporting the presence of H_2_-evolving activity in mitochondria of higher plants (Zhang et al., 2020). In conclusion, the available evidence suggests that eukaryotic mitochondria or MROs initially possessed the capacity to metabolize H_2_, which gradually declined as they adapted to rising oxygen levels in their surroundings; nevertheless, many eukaryotes still retain the ability to metabolize H_2_ today.

**TABLE 2:** The classification of MROs according to Muller et al.’s approach.

Class	MROs	ATP production	H_2_ evolution	ETC	Utilization of O_2_ as the terminal electron acceptor
1	Aerobic mitochondria	Y	N	Y	Y
2	Anaerobic mitochondria	Y	N	Y	N
3	H_2_-producing mitochondria	Y	Y	Y	N
4	Hydrogenosomes	Y	Y	N	N
5	Mitosomes	N	N	N	NA

Abbreviations: Y, yes; N, no; NA, not available.

### 4.2 Complex I shares a common ancestor with group 4 [NiFe]-hydrogenase

**FIGURE 3:**
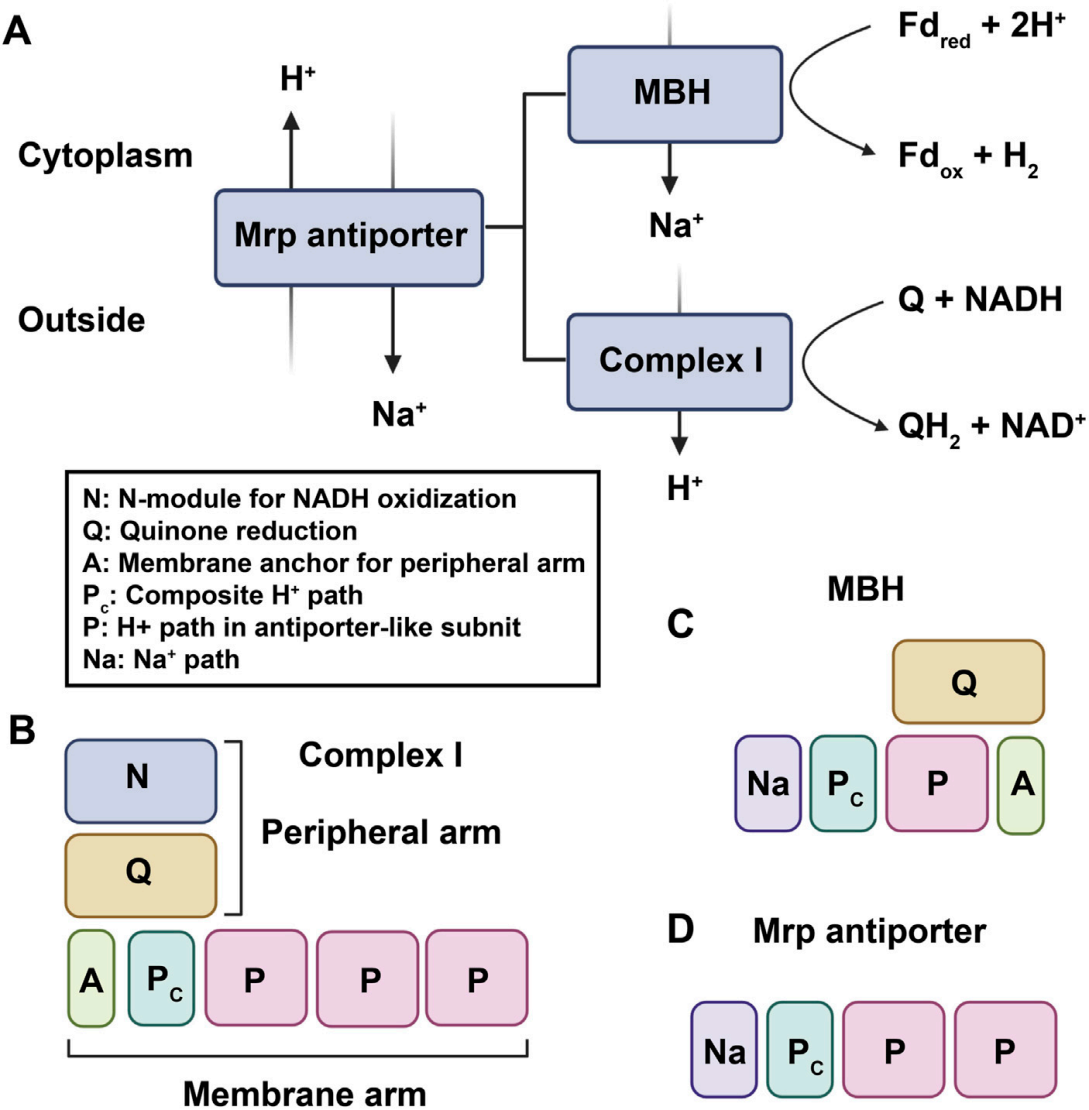
The established evolutionary relationship between MBH, Complex I, and the Mrp antiporter. **(A)**. MBH and Complex I are evolutionarily and functionally related to the Mrp antiporter. Fdred and Fdox represent reduced and oxidized ferredoxin, respectively. **(B–D)**, proposed working models of complex I–like family members: **(B)** complex I; **(C)** MBH; **(D)** Mrp antiporter.

Complex I, also known as NADH: ubiquinone oxidoreductase, is the first and largest enzyme complex of the respiratory chain that contributes approximately 40% of the proton flux for generating proton-motive force during ATP synthesis (Friedrich and Scheide, 2000). The homologues of complex I exist in bacteria, archaea, eukaryotic mitochondria, and plant chloroplasts. Complex I is a unique L-shaped membrane protein comprising a peripheral arm containing iron-sulfur centers and a catalytic site for NADH oxidation, and a membrane arm involved in proton transport. Complex I in bacteria has 14 subunits called NuoA-N (from NADH: ubiquinone oxidoreductase) or Nqo1-14 (from NADH: quinone oxidoreductase), while the mitochondrial enzyme has over 40 subunits. In general, 11 subunits (NuoA to D and NuoH to N) involved in charge translocation and quinone binding are highly conserved across species (Marreiros et al., 2013). Complex I can also be functionally categorized into three modules: the dehydrogenase module (N module, subunits NuoEFG) responsible for accepting electrons from NADH, the hydrogenase module (Q module, subunits NuoBCDI) involved in delivering electrons to ubiquinone, and proton translocating P-module (subunits NuoLMNKAJ). While the N and Q modules belong to the matrix arm, the P module is located within the membrane arm (Sharma et al., 2009). [NiFe]-hydrogenases are categorized into four groups according to the phylogeny of their catalytic subunits. Group 4 [NiFe] hydrogenases distinguish themselves from other groups by incorporating non-hydrogenase components, like carbon monoxide dehydrogenase and formate hydrogenlyase, which facilitate the transfer of electrons from diverse donors to protons, leading to H_2_ production. This electron transfer is coupled with Na^+^ translocation across the membrane, ultimately driving ATP synthesis via Na^+^-driven ATP synthase (Yu et al., 2021). Moreover, Group 4 [NiFe] hydrogenases are notably more complex than dimeric hydrogenases and consist of a minimum of six subunits. Phylogenetic analysis strongly supports the significant evolutionary relationship between the catalytic subunits of Group 4 [NiFe]-hydrogenases and the Q module of Complex I (Schut et al., 2016). One of the best characterized Group 4 hydrogenases are the 14-subunit membrane-bound hydrogenase (MBH) from the hyperthermophilie *Pyrococcus furiosus*. The cryo-EM analysis revealed that both Complex I and MBH possess a closely-related module in their peripheral arm, anchored to a membrane subunit, facilitating the reduction of either protons (MBH) or quinones (Complex I). Moreover, MBH shares a potential proton-translocation module with Complex I, despite their distinct orientation in relation to the peripheral module of each complex (Yu et al., 2018). The significant difference between the two complexes is the presence of a sodium ion translocation unit, absent in Complex I but present in subunit A-C and F of MBH, and also found in the Mrp antiporter (Multiple resistance and pH antiporter) which confers resistance to high Na^+^ stress through an essential H^+^/Na^+^ exchange mechanism in numerous bacteria (Yu et al., 2018). These structural findings validate the established evolutionary relationship between MBH, Complex I, and the Mrp antiporter, providing evidence that they may have evolved through the assembly of pre-existing modules (Figure 3).

### 4.3 H_2_ metabolism in higher plants and animals: potential hydrogenase activity in mitochondria

Besides bacteria and anaerobic/microaerophilic eukaryotes, higher plants and animals have also been reported to possess the capability of H_2_ metabolism, although the evidence from animals is very limited. In 1961, Sanadze firstly reported the activity of both producing and consuming H_2_ in higher plants leaves (Sanadze, 1961). Subsequently, Renwick et al. (1964) demonstrated that various types of germinating higher plant seeds are capable of generating H_2_ both in light and darkness. Since then, the phenomenon of endogenous H_2_ production has been widely reported in higher plants (Torres et al., 1986; Mal’tsev et al., 1988; Xu et al., 2013; Xie et al., 2014; Su et al., 2018; Zhang et al., 2022). Although the photoevolution of H_2_ has been found in subchloroplast preparations of higher plants (Mal’tsev et al., 1988), considering the absence of chloroplast during the early phases of seed germination particularly in the dark, there should be other sources of H_2_ production in higher plants. Animals, in contrast to higher plants, have been scarcely reported to possess the capacity for H_2_ metabolism. In 1962, Kurata reported the presence of hydrogenase activity during the embryonic development of frogs (Kurata, 1962). However, to date, no further experimental evidence has been reported regarding animals exhibiting the capacity to metabolize H_2_.

Taking into account the ability of MROs in eukaryotic microalgae to metabolize H_2_, as well as the homology between eukaryotic complex I and hydrogenase, it is reasonable to propose that the observed production of H_2_ in higher plants might be attributed to the potential hydrogenase activity of mitochondria. In a recent study, Ma’s group presented the initial evidence of H_2_-evolving activity in mitochondria of higher plants, shedding light on the potential contribution of mitochondrial complex I and UQ in H_2_ generation (Zhang et al., 2020). Their findings revealed that H_2_ production significantly increased in hypoxic conditions compared to normoxic conditions, indicating the inhibitory impact of O_2_ on H_2_-evolving activity. The experimental evidence suggests that the accumulation of succinate in mitochondria during hypoxic conditions triggers reverse electron transfer (Chouchani et al., 2014), depleting the UQ pool. As a result, protons can then compete with UQ for electrons, ultimately resulting in the release of H_2_. However, a number of crucial questions remain elucidated, such as the mechanism and catalytic site of mitochondrial H_2_ production, whether mitochondria also possess H_2_ uptake activity, and the physiological significance of the potential hydrogenase activity in higher plants. In addition, the potential existence of hydrogenases in animal mitochondria cannot be ruled out, even though there are very limited reports on H_2_ metabolism in animal cells. Further investigation is needed to explore the possible presence of mitochondrial hydrogenase activity in specific cell types during distinct stages of animal development or in response to special conditions.

## 5 Conclusions and perspectives

To date, substantial experimental evidence has demonstrated that H_2_ exerts diverse biological effects on various organisms, including animals, plants, and intestinal flora. However, the mechanism underlying the action of H_2_ and its target molecules remain unclear, posing an urgent challenge in the field of hydrogen biology research. Increasing evidence suggests that H_2_ can alleviate mitochondrial dysfunction caused by disease or external stress, as summarized in the preceding sections, the mechanisms underlying H_2_-induced mitochondrial responses seem to vary depending on the extent of mitochondrial damage. Although several possible mechanisms have been proposed for the biological effects of H_2_ through the mitochondrial pathway, the current research encounters several challenges. These unresolved issues at least include: 1) despite the potential involvement of mtROS as a target of H_2_, the specific mitochondrial target molecules for H_2_ action remain unclear. Future mechanistic studies should prioritize the identification of potential targets for H_2_ and explore the intricate interactions between H_2_ and these target molecules; 2) further investigation is needed to confirm the potential impact of H_2_ on specific enzyme activity or protein function within mitochondria through PTMs; 3) the impact of H_2_ on the transport of mitochondrial ions (e.g., Ca^2+^, Na^+^, and K^+^) and its possible mechanisms; 4) the potential hydrogenase activity of eukaryotic mitochondria and its physiological significance; 5) the effects of H_2_ on mitochondria under normal conditions. The future research on the mechanism of H_2_ action may be accelerated through the application of advanced technologies such as stable-isotope labeling, cryo-electron microscopy, and single-cell and spatial multi-omics.

In conclusion, preliminary experimental evidence has shed light on the role of H_2_ through the mitochondrial pathway. However, future research endeavors must focus on more comprehensive and detailed investigations to unravel the remaining unanswered questions in this field. It is anticipated that elucidating the mitochondrial mechanism of H_2_ will not only unravel the mystery of the mechanism of H_2_ action but also potentially provide additional experimental evidence for the study of eukaryotic evolution.

## Figures and Tables

**Table:** 

**H**_**2**_	molecular hydrogen
**ROS**	reactive oxygen species
**I/R**	ischemia-reperfusion
**HRW**	hydrogen-rich water
**HRS**	hydrogen-rich saline
**ATP**	adenosine triphosphate
**NSC**	no significant change
**mPTP**	mitochondrial permeability transition pore
**MMP**	mitochondrial membrane potential
**mtROS**	mitochondrial reactive oxygen species
**NA**	not available
**mtDNA**	mitochondrial deoxyribonucleic acid
**mMDA**	mitochondrial malondialdehyde
**mGSSG**	mitochondrial glutathione disulfide
**mGSH**	mitochondrial glutathione
**mSOD**	mitochondrial superoxide dismutase
**mCAT**	mitochondrial catalase
**mGpx**	mitochondrial glutathione peroxidase
**MFN1/2**	mitofusin 1/2
**OPA1**	optic atrophy 1
**Drp1**	dynamin-related protein 1
**VDAC1**	voltage dependent anion channel 1
**mtUPR**	mitochondrial unfolded protein response
**mitoKATPs**	mitochondrial ATP-sensitive potassium channel
**OVA**	ovalbumin
**ALI**	acute lung injury
**ETC**	electron transport chain
**PARP-1**	poly (ADP-ribose) polymerase-1
**PINK1**	PTEN induced putative kinase 1
**LC3-II**	microtubule-associated protein light chain 3-II
**PGC-1**	peroxisome proliferator-activated receptor-gamma (PPARγ) co-activator-1alpha
**TFAM**	mitochondrial transcription factor A
**NRF1/2**	erythroid 2-related factor 1/2
**PPARγ**	peroxisome proliferator-activated receptor-gamma
**DCFH-DA**	edichloro-dihydro-fuorescein diacetate
**FLP**	frustrated Lewis pair
**UQ**	ubiquinone
**2-OH-E** ^**+**^	2-hydroxyethidium
**HO-1**	heme oxygenase-1
**NQO1**	NAD(P)H quinone dehydrogenase 1
**PCD**	programmed cell death
**Bcl-2**	B-cell lymphoma 2
**Bax**	Bcl-2-associated X protein
**TUNEL**	terminal deoxynucleotidyl transferase dUTP nick end labeling
**PI**	propidium iodide
**AIF**	apoptosis-inducing factor
**ARE**	Nrf2/antioxidant responsive element
**SIRT1**	Sirtuin1
**PI3K**	phosphatidylinositol 3′-kinase
**Foxo3a**	forkhead box O3
**VSMCs**	vascular smooth muscle cells
**AMPK**	AMP-activated protein kinase
**SMC3**	structural maintenance of chromosomes 3
**STAT3**	signal transducer and activator of transcription 3
**ERK1/2**	Ras-extracellular-regulated protein kinases 1/2
**MEK1/2**	mitogen-activated or extracellular signal-regulated protein kinase kinases 1 and 2
**OMM**	outer mitochondrial membrane
**LPS**	lipopolysaccharide
**CLP**	cecal ligation and puncture
**PTMs**	post-translational modifications
**Ub**	ubiquitin
**FUNDC1**	FUN14 domain containing 1
**eIF2α**	eukaryotic translation initiation factor 2 subunit 1
**5′UTR**	5′untranslated region
**CHOP**	C/EBP homologous protein
**ATF4**	transcription factor 4
**mtHsp70**	mitochondrial heat shock protein 70
**FGF21**	fibroblast growth factor 21
**5-HD**	5-hydroxydecanoate
**mPT**	mitochondrial permeability transition
**MPG**	N-2-mercaptopropionylglycine
**GHS-R1α**	growth hormone (GH) secretagogue receptor 1α
**GLUT1**	glucose transporter 1
**TCA**	tricarboxylic acid
**MROs**	mitochondrion-related organelles
**PFO**	pyruvate: ferredoxin oxidoreductase
**Fd**	ferredoxin
**Nuo**	NADH: ubiquinone oxidoreductase
**Nqo**	NADH: quinone oxidoreductase
**MBH**	membrane-bound hydrogenase
**Mrp antiporter**	multiple resistance and pH antiporter
